# mTOR Signaling in Macrophages: All Depends on the Context

**DOI:** 10.3390/ijms26157598

**Published:** 2025-08-06

**Authors:** Angelika Fedor, Krzysztof Bryniarski, Katarzyna Nazimek

**Affiliations:** Department of Immunology, Jagiellonian University Medical College, Czysta 18, 31-121 Kraków, Poland; angelika.fedor@uj.edu.pl (A.F.); krzysztof.bryniarski@uj.edu.pl (K.B.)

**Keywords:** autoimmunity, immune regulation, immune tolerance, macrophages, mammalian target of rapamycin, mechanistic target of rapamycin, miRNAs, miRs, mTORC1, mTORC2

## Abstract

Macrophages are undoubtedly one of the most widely studied cells of the immune system, among other reasons, because they are involved in a wide variety of biological processes. Deregulation of their activity is observed in a number of different disorders, including autoimmune diseases. At the same time, mammalian target of rapamycin (mTOR) is attracting increasing research attention because the pathways dependent on this kinase are activated by a variety of signals, including cytokines and proinflammatory mediators, mediate essential processes for cell survival and metabolism, and can be regulated epigenetically via microRNAs. Therefore, our narrative review aimed to summarize and discuss recent advances in the knowledge of the activation of mTOR signaling in macrophages, with a special focus on autoimmune disorders and the possibility of mTOR control by microRNAs. The summarized research observations allowed us to conclude that the effects of activity and/or inhibition of individual mTOR complexes in macrophages are largely context dependent, and therefore, these broad immunological contexts and other specific conditions should always be taken into account when attempting to modulate these pathways for therapeutic purposes.

## 1. Introduction

Narrative reviews aim to combine multiple findings from individual studies into a single global picture. This is similar to stargazing, which is affected by time of night and season, latitude, and confounding factors, but when coherent, allows for the discovery of all the constellations of stars within the range of the most sensitive telescopes. From a biological perspective, the use of increasingly precise and sensitive methods has opened completely new horizons in medical research, revealing that even a single protein participates in many different signaling pathways, and the biological effects of its activation/inhibition are determined in a context-dependent manner. This progress also applies to current research in medical immunology, proving the complexity of the cellular and molecular mechanisms controlling the body’s immunity. In this respect, macrophages are an excellent example of the complexity and balance of cellular processes that contribute to their diverse functions and activation phenotypes. As a result, macrophage activity is tailored to maintain tissue homeostasis, and its disruption results in serious inflammatory complications and the development of various diseases.

Macrophages are a part of the innate immune system and are found in large numbers in all mammalian tissues, constituting the body’s first line of defense against pathogens and tumor cells [1,2,3,4]. As their monocyte precursors, they belong to the mononuclear phagocyte system (MPS), along with dendritic cells, and they are essential in phagocytosis, immunomodulation, and antigen presentation [5]. Additionally, they play a key role in controlling immune processes and maintaining the body’s homeostasis [2]. It is nowadays widely accepted that tissue-populating macrophages originate from the precursors that mature in the yolk sac (most of the resident macrophages) or in the bone marrow (infiltrating macrophages and some resident counterparts) [4]. It should be emphasized that macrophages can quickly shift their phenotype in response to the prevailing state of the local microenvironment and the acting stimuli. This feature allows macrophages to perfectly adapt to the current tissue conditions. Therefore, they constitute a heterogeneous population of immune cells that are generally divided into M1 and M2 phenotypes, although the current activation state of macrophages usually expresses a very specific intermediate phenotype. Nonetheless, classically activated M1 macrophages are characterized by proinflammatory and cytotoxic features and multiply inflammatory signaling. They polarize towards the M1 phenotype in response to Th1 lymphocyte cytokines (tumor necrosis factor alpha, TNFα, and interferon gamma, IFNγ) as well as extra- and intracellular pathogens and their components, i.e., ligands of pattern recognition receptors (PRRs), such as Toll-like receptors (TLRs), that are collectively called pathogen-associated molecular patterns (PAMPs), of which lipopolysaccharide (LPS) is the most studied example. Conversely, alternatively activated M2 macrophages have anti-inflammatory properties, and thus participate in inhibiting the inflammatory processes and promoting tissue regeneration, but they are also involved in immune suppression that promotes the growth of cancer cells. M2 macrophages polarize in the presence of type II cytokines, such as IL-4 and IL-13, as well as a combination of other factors, including IL-10, TGFβ, immune complexes, some TLR ligands, glucocorticoids, and macrophage colony-stimulating factor (M-CSF) [6,7]. Growing evidence supports that multiple molecular pathways control macrophage activity, of which the mammalian target of rapamycin (mTOR) signaling appears to be the most complex and context-dependent, especially since it links immunological, metabolic, and growth factor/hormonal pathways [8].

Although macrophages have been very well studied so far, there is always something important to add, because these cells can engage in almost any biological process owing to their extraordinary adaptability. Therefore, our narrative review summarizes the current knowledge on the multifaceted role of mTOR signaling in macrophages to highlight the complex interactions between molecular aspects of macrophage immunological, biological, and metabolic functions.

## 2. A Multifaceted Role of mTOR Signaling in Macrophages

The extraordinary plasticity of the macrophage phenotype and its ability to quickly adapt to the current microenvironmental conditions are governed by several molecular pathways [2] and depend, among other factors, on rapid metabolic reprogramming. Because mTOR is a key regulator of cellular metabolism, it is increasingly evident that macrophages can specifically switch between different mTOR-related pathways to best match their activation status to the current demand. Conversely, modulation of the mTOR signaling cascades actively shapes the macrophage phenotype and function. Therefore, it is crucial to investigate the interactions of mTOR pathways in macrophage biology to better understand the observed complex immunological effects.

### 2.1. mTOR—The Central Kinase Linking Cellular, Immune, and Metabolic Pathways

**Figure 1:**
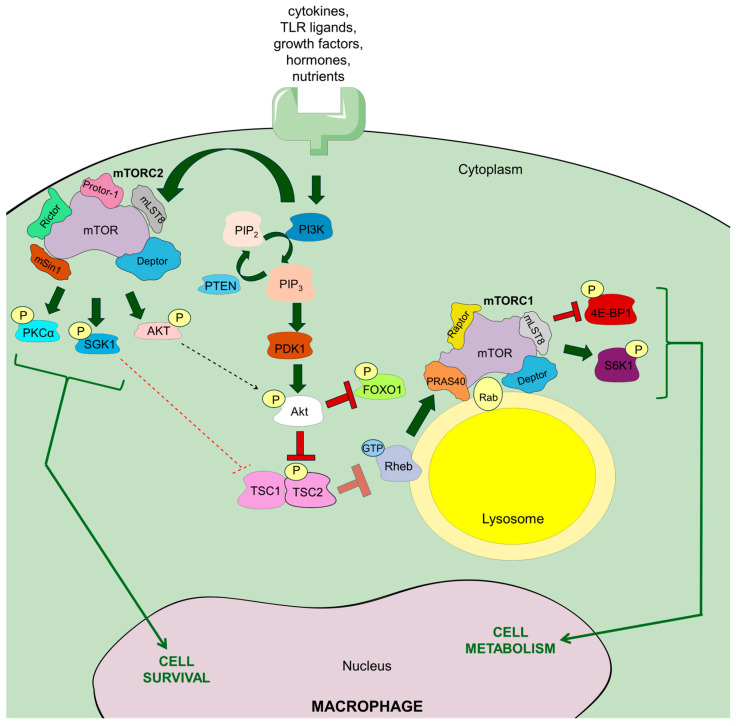
The general scheme of mTOR complexes (mTORC1 and mTORC2) as well as signaling pathways in macrophages. Receptor stimulation by a variety of signaling molecules and nutrients enables binding of phosphatidylinositol 3-kinase (PI3K), which in turn generates phosphatidylinositol 3-phosphate (PIP_3_/PtdInsP3), but dephosphorylation of PtdInsP3 via phosphatase and tensin homolog (PTEN) may block this process. PIP_3_ generation is followed by the activation of phosphoinositide-dependent protein kinase 1 (PDK1), which phosphorylates Akt kinase. Phosphorylated Akt further phosphorylates tuberous sclerosis complex 2 (TSC2), which prevents the blockage of mTORC1 formation. Precisely, it enables Ras homolog enriched in brain (Rheb) to bind GTP and stimulate mTORC1 formation in the lysosome membrane. PI3K activation also allows for formation of mTORC2 in the plasma membrane, and mTORC2 phosphorylates Akt, which may, in turn, drive mTORC1 formation. Apart from Rheb, mTORC1 trafficking to the lysosomal membrane is also supported by Rab GTPases, especially Rab4A. Arrow explanation: bold green—activation; bold red—inhibition; bold red transparent—blocked inhibition; thin dashed green—postulated positive feedback loop; thin dashed red—postulated negative feedback loop; thin green—main cell effects.

mTOR (also known as mechanistic target of rapamycin) is an enzyme from the serine-threonine kinase group, belonging to the PI3K-related kinase (PIKK) family. It performs a catalytic function in two different protein complexes, namely mTORC1 (mammalian target of rapamycin complex 1) and mTORC2 (mammalian target of rapamycin complex 2), which have different roles in regulating cellular processes [9]. Mechanistically, mTORC1 was identified as a multi-protein complex that includes mTOR, along with Raptor (a regulatory protein that associates with mTOR), mLST8 (mammalian lethal with Sec13 protein 8, also known as GβL, a protein similar to the G protein β subunit), DEPTOR (a protein with a DEP domain that interacts with mTOR), and PRAS40 (a 40 kDa protein that acts as a proline-rich substrate of Akt) [9,10]. mTORC2 is a protein complex made up of mTOR, Rictor (a partner of mTOR that is not sensitive to rapamycin), mLST8 (GβL), DEPTOR, mSIN1 (mammalian stress-activated protein kinase-interacting protein-1), and Protor (PRR5, proline-rich protein-5) [10]. It is worth noting that mTORC1 is anchored in the lysosomal membrane, whereas mTORC2 is formed in the plasma membrane, which to some extent determines their functional activity (Figure 1) [11]. In addition, some studies report that mTORC1 can be formed in the plasma membrane, endoplasmic reticulum, Golgi apparatus, and nucleus [12], while mTORC2 can localize to mitochondria and a subtype of endosomal vesicles [13].

Moreover, differences in the composition of both complexes determine different substrate preferences and responses to rapamycin. Exactly, rapamycin effectively and quickly inhibits mTORC1, while mTORC2 is only partially affected, and usually only after extended treatment [14]. mTOR plays a key role in numerous biological functions, such as cell growth, survival, immune response, autophagy, and metabolic regulation [10]. More specifically, mTORC1 is an extremely sensitive sensor of nutrient availability and primarily oversees cell metabolism and growth, whereas mTORC2 is responsible for cell survival and proliferation [15]. mTOR has been shown to play an important role in various signaling pathways, such as PI3K/Akt (phosphatidylinositol 3-kinase/Akt) pathway, the LKBL/AMPK pathway, the TSC1/TSC2/Rheb system (a protein complex associated with tuberous sclerosis), as well as processes dependent on VAM6 and Rag GTPases, and many others [15]. Furthermore, growth factors, hormones, amino acids (mostly arginine and leucine, but also glutamine [16]), cellular stress (reactive oxygen species—ROS, DNA damage), and energetic substrates (glucose especially) are the main upstream regulators of mTORC1, which modulates various biological processes, such as the synthesis of lipids, nucleotides and proteins, autophagy and mitochondrial biogenesis. On the other hand, the main upstream regulators of mTORC2 are growth factors that control cell migration and proliferation, ion transport, glucose metabolism, or cytoskeletal remodeling [10].

In the case of the innate immune cells, mTORC1-mTORC2 signaling is stimulated by various extracellular signals, including cytokines, growth factors, and TLR ligands (Figure 1) [17]. In neutrophils and dendritic cells, granulocyte/macrophage colony-stimulating factor (GM-CSF) and FMS-related tyrosine kinase 3 ligand (FLT3L) induce mTORC1 formation. Activation of mTORC1 and mTORC2 occurs via TLR ligands in human and mouse monocytes, macrophages, and dendritic cells. In contrast, only mTORC1 seems to be activated in mouse neutrophils via TLR signaling. Furthermore, activation of mTORC1 and mTORC2 occurs via IL-4 in mouse macrophages and via IL-15 in human and mouse NK cells. Although the exact pathway of mTORC2 activation remains unclear, there is growing evidence that similar growth factors can influence this pathway through PI3K [18,19], and recent observations indicate that mTORC2 can also be activated by insulin, phosphatidylinositol-3,4,5-triphosphate (PtdInsP3), and enhanced plasma membrane tension through phospholipase D2 (PLD2) [20,21]. mTORC1 and mTORC2 signaling appear to be firmly reciprocally related [18]. This is especially seen during mTORC1 induction, when, in response to insulin and insulin-like growth factor 1 (IGF1), mTORC2 complex phosphorylates Akt, the crucial upstream regulator of mTORC1, at serine 473, which appears to be essential for its full activation and substrate specificity [17]. Conversely, mTORC2 activation can be restricted by mTORC1-S6K1 signaling in macrophages [20].

When nutrients are readily available, the Ragulator complex of five late endosomal/lysosomal adaptor molecules (Lamtor1-5) tethers Rag GTPases to the lysosomal membrane, and together with v-ATPase allows for amino acid-driven formation of mTORC1 [22]. More specifically, in the case of mTORC1 activation in macrophages, amino acids, delivered via micropinocytosis (see below) [23], provoke coupling of Rag proteins to Raptor, which promotes the relocalization of mTORC1 with Rheb-GTP, leading to its activation. In the case of mTOR activation by growth factors, cytokines, and PAPMs, their binding to the corresponding receptors, TLRs especially, recruits PI3K kinase via the small GTPase Rab8A, which is typical for macrophages [10,17,24]. When bound to the respective receptor, PI3K generates PtdInsP3. This is followed by the phosphorylation at threonine 308 and activation of the serine-threonine Akt kinases (namely Akt1, Akt2, and Akt3) via phosphoinositide-dependent protein kinase 1 (PDK1). It is worth mentioning that dephosphorylation of PtdInsP3 via phosphatase and tensin homolog (PTEN) may negatively regulate this process. Akt phosphorylated at threonine 308 (by PDK1) and serine 473 (by mTORC2, see above) phosphorylates TSC2 (tuberous sclerosis complex-2). Under steady-state conditions, TSC2 and TSC1 form a complex that inhibits mTORC1 activation. However, TSC2 phosphorylation at threonine 1462 leads to inhibition of its GTPase-activating protein (GAP) activity towards Rheb (RAS homolog enriched in brain), thereby enabling Rheb to bind GTP and stimulate mTORC1 formation in the lysosome membrane via phospholipase D1 (PLD1) and suppression of FKBP38. In parallel, activation of mTORC1 can also be mediated by the mitogen-activated protein kinases (MAPKs) p38α and COT, inducing MK2 (mitogen-activated protein kinase-activated protein kinase-2) and ERK (extracellular signal-regulated kinase), respectively. Consequently, both MK2 and ERK phosphorylate TSC2, which enables mTORC1 activation [17,25]. The existence of some prerequisites for mTORC1 activation via these pathways is also worth highlighting. Namely, lysosomal amino acid availability, sensed by Rag GTPases, has an activating effect on mTORC1 signaling by growth factors, similarly to the direct binding of phosphatidic acid, a crucial intermediate in lipid metabolism, to mTORC1 [17]. Finally, increased glucose intake stimulates PLD1 to generate phosphatidic acid, which is required for the formation of the mTORC1 complex [25].

Conversely, under low cellular energy levels, sensed as high AMP concentrations by AMP-activated protein kinase (AMPK), this kinase phosphorylates TSC2 at serine 1387 and also Raptor, which prevents mTORC1 activation through the TSC1-TSC2 complex. Conversely, mTORC1 has recently been shown to negatively regulate AMPK [26], which proves the importance of AMPK-mTORC1 reciprocal balancing in cellular metabolism. mTORC1 is also blocked when bound by hexokinase-2 (HK2), which is promoted by low levels of glucose-6-phosphate [17].

The downstream effects of mTORC1 activation result from its catalytic activity against specific substrates. Specifically, mTORC1 phosphorylates and activates ribosomal protein kinase S6 type 1 (S6K1 or also called RPS6KB1), which plays an important role in protein synthesis and nucleotide availability (together with ATF4). The protein synthesis is also promoted by mTORC1-mediated phosphorylation of translation inhibitors, such as eIF4E-binding protein 1 (4E-BP1) and 4E-BP2, which release eukaryotic translation initiation factor 4E (eIF4E) that acts as a cap-binding protein initiating mRNA translation. Furthermore, mTORC1 phosphorylates TFEB and ULK1, which inhibits autophagy. Finally, mTORC1 enzymatic activity against TFEB, as well as SERBPs and Lipin1, regulates lysosomal biogenesis and lipid synthesis, respectively [10].

Apart from Akt, mTORC2 phosphorylates protein kinase C (PKC), as well as serum- and glucocorticoid-regulated kinase 1 (SGK1), resulting in the regulation of various cellular processes, including ion transport, cell survival, proliferation, and migration along with cytoskeletal reorganization [10]. Interestingly, phosphorylated Akt targets forkhead box O1 (FOXO1) [17], which contributes to the modulation of macrophage-dependent cytokine circuits, especially by downregulating IL-12 synthesis, as discussed below.

These complex upstream and downstream mTOR signaling pathways induce a range of biological effects in macrophages that have a significant impact on homeostasis and overall body functioning.

### 2.2. mTOR in Macrophage Differentiation and Polarization

Circulating monocytes originate from hematopoietic stem cells in the bone marrow, passing through the monocyte/dendritic cell precursor stage, and then differentiating into macrophages upon entry into inflamed tissues. These monocyte-derived macrophages play a crucial role in immune defense, while tissue-resident macrophages, mostly originating from the yolk sacs or fetal liver-derived progenitors, are primarily responsible for tissue homeostasis [27]. In the early stages of myelopoiesis, mTOR deficiency leads to overstimulation of STAT5 signaling, which reduces the activity of IRF8, a transcription factor critical for monocyte differentiation in the bone marrow [28]. Subsequent studies on myeloid lineage differentiation have also provided important insights into the role of mTOR signaling in macrophage maturation [29]. Specifically, M-CSF stimulation of myeloid precursors triggers mTORC1-induced anabolic reprogramming to activate sterol biosynthesis and the Myc transcriptional pathway, which increases M-CSF receptor expression to drive myelopoiesis [30]. This myelopoiesis feedback loop is controlled by the S6K1 pathway, downstream of mTORC1, that represses Myc activity, which is required for terminal myeloid differentiation [31]. Of note, mTORC1 activity measured as S6 phosphorylation, a functional readout of S6K1 activity, decreases during myeloid cell differentiation, reaching the lowest value in mature Ly6C^lo^ monocytes [31]. However, simultaneous inhibition of PPARγ and mTORC1 results in GM-CSF-directed monocyte differentiation towards dendritic cells [32], suggesting that low but sustained mTORC1 activity in monocytes is necessary for their final maturation towards macrophages. On the other hand, constitutive overactivation of mTORC1 caused by TSC1 deficiency leads to a decrease in the number of alveolar macrophages due to their reduced survival and impaired responsiveness to IL-13 [33]. Conversely, under homeostatic conditions, the embryonic precursor-derived alveolar macrophage population exhibits a high self-renewal potential [34] that depends on mTORC1 activity, enabling GM-CFS-induced proliferation [35]. Similarly, ceramide 1-phosphate-driven macrophage proliferation was also found to be mTORC1-dependent [36]. GM-CSF has been found to drive macrophage differentiation from mouse embryonic stem cells by activating the mTOR signaling pathway, thereby preventing autophagy [37]. In contrast, autophagy plays a crucial role in protein digestion during monocyte-to-macrophage transition, and its blockage by mTORC1 overactivation diminishes monocyte differentiation towards mature macrophages [38], whereas mTOR-driven promotion of macrophage differentiation could be induced by fibronectin leucine-rich transmembrane protein 2 [39]. Finally, increasing evidence points to the involvement of mTOR pathways in macrophage senescence [40]. Therefore, their inhibition may have therapeutic potential by mitigating the inflammatory consequences of immune senescence [41]. It is also worth noting that, in certain circumstances, mTOR signaling prompts monocyte differentiation towards M2 macrophages [42], similarly to TSC2 knockdown [43], while monocyte-to-macrophage transition is blocked in Rheb1-deficient mice [44]. At an earlier stage of hematopoiesis, ligation of the CD137 receptor drives a monocyte/macrophage differentiation program in their progenitors [45], which seems to involve the mTOR pathway by increasing responsiveness to M-CSF [46]. It is also worth mentioning that mTORC1 activity regulates microglia, Kupffer cells, osteoclast differentiation, and tissue-specific functions [47,48,49,50,51].

Furthermore, low mTORC2 activity is a characteristic feature of tissue-resident macrophages and distinguishes them from monocyte-derived counterparts in the mouse peritoneal cavity [52]. This may be due to the fact that mTORC2 signaling is necessary for the efficient migration of monocytes to inflammatory sites and their full maturation into macrophages [53]. Similarly, the mTORC2 pathway plays a pivotal role in macrophage polarization towards the M2 phenotype [54], and Rictor-deficient macrophages are not only unable to alternatively activate but also exhibit reduced migratory and tissue repair activity [55] as well as diminished self-renewal [56]. These findings strongly imply an important role of the mTORC2 complex in maintaining homeostatic functions of tissue-resident macrophages. Additionally, the neuropeptide substance P perpetuates tissue-repairing M2 phenotype in macrophages by activating the PI3K/Akt/mTOR pathway [57]. Conversely, observations regarding the role of mTORC1 activity in macrophage polarization are ambiguous [58,59]. While constitutive activation, acute inflammation (e.g., after myocardial infraction) and infectious conditions seem to reveal a promoting role of mTORC1 in M1 macrophage polarization [60,61,62,63], other studies demonstrated that rapamycin treatment [64] and genetic deletion of mTORC1 molecular components favors M1 phenotype without any significant changes in glucose metabolism in mouse macrophages [65]. The latter observations somewhat disrupt the paradigm that macrophage polarization is closely linked to the metabolic switch, i.e., that the proinflammatory phenotype is associated with increased glycolysis, whereas M2 activation is based on oxidative phosphorylation. This seems likely because, upon inflammatory stimulation, the Warburg effect in macrophages is differently regulated by the PI3K–Akt–mTOR axis [66]. Conversely, existing data showed that the functional Lamtor1 component of Ragulator is required for macrophage M2 polarization in response to simultaneous cytokine and amino acid signaling [67]. Regardless of this, based on their detailed molecular research findings, Byles and colleagues proposed that, in most cases, mTORC1 activation supports M2 polarization, whereas the M1 phenotype is promoted after LPS stimulation when mTORC1 is chronically activated under nutrient-rich conditions [60]. Accordingly, some preliminary data imply that mTORC1 activity is essential for M2 polarization in acute LPS-induced endotoxemia in mice [68]. Furthermore, recent reports suggest that mTORC1 downstream signaling via S6K1 promotes the M2 phenotype in mouse testicular macrophages under the influence of insulin-like peptide 3 (INSL3), largely produced by Leydig cells [69]. Similarly, PI3K/Akt/mTOR activated by Liver X receptor α (LXRα) agonist diminished M1 polarization of macrophages, thereby protecting rats against liver injury after organ transplantation [70]. On the other hand, overexpression of Castor1, an upstream regulator of the mTOR cascade, promoted M2 polarization of mouse microglial cell line by reducing mTORC1 activity [71], and similar outcomes can be achieved by rapamycin treatment [72]. Thus, M2 microglia polarization induced by mTORC1 inhibition exerts neuroprotective effects [73,74], but also a deleterious effect in glioblastoma [75,76,77,78,79]. Additionally, mTOR signaling confers proper phagocytic activity of microglia, which is critical to maintain neuronal homeostasis by removing dead neurons and synapse pruning [80]. Mechanistic studies revealed that cyclic GMP-AMP synthase (cGAS), which senses cytosolic DNA, skews macrophage polarization towards the M1 phenotype by inducing mTORC1 signaling via mitochondrial DNA [62]. Overall, these findings imply a complex role for mTOR signaling in macrophage polarization to ensure immune quiescence in immune-privileged tissues.

It should be stressed that macrophage M2 polarization may have a deleterious effect. Accordingly, Rheb1-deficient mice with impaired mTORC1 pathway develop exacerbated allergic asthma even though their alveolar macrophages exhibit an M2 phenotype [81]. Whereas alternative polarization of tumor-associated macrophages (TAMs) expressing upregulated mTORC1 activity promotes glioma progression [82]. Furthermore, oncostatin M promotes M2 phenotype in TAMs via mTORC2 activation, which favors breast cancer growth and metastatic potential [83]. Similarly, phosphoglycerate dehydrogenase-mediated serine biosynthesis triggers α-ketoglutarate production, which in turn activates mTORC1 signaling in TAMs to maintain their M2 phenotype [84]. Of note, programmed death ligand 1 (PD-L1) seems to promote macrophage polarization towards M2 phenotype via Erk/Akt/mTOR pathway [85]. Thus, once again, it all depends on the context.

In infectious conditions, some pathogens induce M2 macrophage polarization to increase their survival. This effect was proved to depend on the PI3K/Akt/mTOR pathway, for instance, in echinococcosis [86,87], leishmaniosis [88,89], as well as tuberculosis [90]. However, the survival of *Leishmania donovani* within macrophages was favored by mTOR inhibition [91]. In addition, activation of mTOR and PPARγ signaling pathways in macrophages infected with *Trypanosoma cruzi* ameliorates vascular complications caused by Chagas disease [92], but can also promote intracellular survival of *Mycobacterium tuberculosis* [93], whereas *Treponema pallidum* was found to stimulate the M1 polarization of THP-1 macrophages, and this effect was augmented by rapamycin [94]. Finally, inflammatory overactivation of macrophages is a hallmark of some infectious diseases and may be triggered by mTOR signaling [95,96,97].

Interesting studies involving patients with major depressive disorder revealed an increase in SGK1 and FOXO1 expression following polarization of monocyte-derived macrophages towards the M2 phenotype with IL-4 or dexamethasone, and similar results were observed for ketamine-stimulated macrophages, whereas combined treatment with LPS and IFNγ elevated SGK1 but not FOXO1 levels [98]. Mechanistically, the simultaneous increase in SGK1 and FOXO1 levels is quite surprising because mTORC2 activity does not appear to affect total cellular levels of FOXO1 but instead controls its phosphorylation, nuclear localization, and transcriptional activity [99].

From another point of view, observations made in studies on the metabolic syndrome have led to the hypothesis that mTORC1 can be activated by both M1 and M2 macrophage phenotype-promoting factors, i.e., LPS, IFNγ, TNFα, IL-4, and IL-13, respectively, resulting in completely different metabolic effects. Namely, mTORC1 promotes HIF-1α-driven aerobic glycolysis in M1 macrophages and PPARγ signaling along with fatty acid oxidation in M2 macrophages [59]. However, high concentrations of fatty acids have been proposed to polarize macrophages towards the M1 phenotype by increasing mTOR signaling, thus reducing autophagy [100]. Otherwise, IL-4 has been shown to divert Akt/mTORC1 signaling towards the regulation of ATP-citrate lyase (Acly), a key enzyme in the acetyl coenzyme A synthesis pathway, thus calibrating macrophage metabolism to the energetic requirements of M2 phenotype activation [101]. In parallel, M-CSF signaling has been shown to act synergistically with IL-4 to activate the mTORC2 pathway, which in turn directs metabolic reprogramming in M2 macrophages in an IRF4-dependent manner [102]. Intriguingly, modulation of mTOR pathways in tissue-resident macrophages may have different biological outcomes depending on macrophage location [29]. It can therefore be concluded that the effects induced in macrophages by mTOR pathways depend on the immunological, tissue microenvironmental, and metabolic context [103].

### 2.3. mTOR—A Master Regulator of Macrophage Activity

As mentioned above, mTOR kinase forms two distinct complexes, namely mTORC1 and mTORC2, by attaching specific adapter proteins that target it to the lysosomal or plasma membrane, respectively [11]. In the case of differentiated macrophages, mTORC1 signaling can be induced by both cellular/immune and metabolic factors, including growth factors, hormones, cytokines, TLR ligands, and nutrients, particularly amino acids [17,24]. It is worth noting that mTORC1 activation prompts macrophages towards enhanced glucose consumption [104]. In contrast, the signals that activate mTORC2 are less well understood. Nutrients and growth factors also affect mTORC2 activation, but the context in which they modulate mTORC2 appears to be much more complicated than for mTORC1 [105].

Furthermore, mTORC1 formation in the lysosome membrane can be activated by the delivery of extracellular amino acids into lysosomes via macropinocytosis. Among other factors, this process is induced in macrophages by M-CSF and chemokines, especially CXCL12 [23]. Some studies also suggest that a similar process is involved in the formation of mTORC2 in the plasma membrane of macrophages [106]. Remarkably, rapamycin has been demonstrated to inhibit macropinocytosis of antigens by dendritic cells [107], which implies that mTORC1 promotes this process to drive its own activity. From a clinical point of view, persistent mTORC1 activation in monocytes/macrophages contributes to the pathogenesis of macrophage activation syndrome, a life-threatening complication of autoimmune/autoinflammatory rheumatic diseases [108,109]. However, the stimulating factors determine the effects of mTORC1/mTORC2 activation in macrophages, as summarized below.

#### 2.3.1. mTOR Pathways in the Context of TLR and Cytokine Signaling in Macrophages

With the discovery of the important role of mTOR in immunoregulation, scientific interest in this aspect has increased significantly, including attempts to characterize the role of mTOR in the modulation of macrophage functions. One of the first studies that linked the mTOR pathway with macrophages showed that LPS stimulation induces p70 S6 kinase in RAW macrophages, and rapamycin pretreatment inhibits NO production [110], which suggested a role for p70 S6 kinase, which acts downstream of the mTORC1, in controlling LPS-stimulated NO secretion. This was rapidly confirmed in a subsequent study, identifying mTOR as the crucial inductor of p70 S6 kinase modulatory activity on NO production [111]. Simultaneously, upregulation of genes encoding mTOR-related mediators was shown in LPS-stimulated macrophages [112]. Additionally, rapamycin was suggested to stimulate proteasomal degradation of inducible nitric oxide synthase (iNOS) [113] and reduce G-CSF protein levels in RAW cells [114]. In parallel, a microarray-based approach identified that mTOR inhibition with rapamycin overstimulates the regeneration of matrix metalloproteinase-9 (MMP9) by LPS-treated RAW macrophages, and that mTOR negatively regulates nuclear factor kappa B (NF-κB) and MAPK signaling [115]. Next, mTOR signaling was proposed to control the LPS-stimulated translation of cytokine-encoding genes in macrophages in a 4E-BP1 and FRAP-dependent manner [112,116]. Subsequent studies refined these hypotheses by demonstrating that the mTOR pathway directly regulates the synthesis of IL-6 and IL-10 but not TNFα in LPS-treated human peripheral blood mononuclear cells (PBMCs) [117]. Moreover, pharmacological targeting of mTOR with rapamycin has been shown to block IL-10 synthesis by LPS-stimulated mouse peritoneal macrophages, which in turn increased TNFα production [118]. Interestingly, a similar relationship was observed in alveolar macrophages collected from human immunodeficiency virus (HIV)-infected individuals [119]. Later, the enhancing effect of mTORC1 on IL-10 release was linked with STAT3 phosphorylation and PDCD4/Twist2/c-Maf network [29,120]. Additionally, human umbilical mesenchymal stem cell-derived stanniocalcin-1 upregulates IL-10 in alveolar macrophages by activating the PI3K/Akt/mTOR pathway [121]. Activation of the mTOR pathway has been shown to sensitize macrophages to TNFα-mediated killing in anthrax infection [122]. However, some researchers propose that mTOR signaling biases the pattern of cytokine secretion by macrophages under inflammatory conditions. Namely, mTORC1-driven increase in protein synthesis, which is a hallmark of proinflammatory macrophage activation, is suggested to favor the translation of mRNAs encoding anti-inflammatory cytokines, IL-10 especially, at the expense of proinflammatory cytokine synthesis [19]. Moreover, in LPS-stimulated macrophages, mTORC2 phosphorylates Akt, which in turn limits the activity of the transcription factor FOXO1, reducing the synthesis of IL-12, and this inhibitory effect is reversed by inactivation of mTORC2 [17]. Thus, these bystander effects may partially explain some of the proinflammatory results of mTOR inhibition [19]. Conversely, in certain conditions, signaling through PI3Kγ/Akt/mTor pathway, apart from NF-κB inhibition, stimulates C/EBPβ transcription factor, which promotes immune suppressive macrophage phenotype [123]. This signaling route, similarly to the IL-10-enhancing effect of the PI3K/Akt/mTOR pathway in monocytes/macrophages [124], is used by tumor cells to promote immune tolerance and maintain M2 phenotype in TAMs [6]. Analogous impact has been shown in the case of gut microbiota-stimulated cathepsin K that induced M2 phenotype in TAMs by binding to TLR4 and activating the mTOR axis [125]. However, some studies reported that sustained mTORC1 activity caused by genetic deletion of TSC1 in TAMs induces their pro-resolving phenotype, due to which they rectify a hyperpermeable endothelium, causing severe hypoxia and tumor cell death [126]. Meanwhile, mTOR induction in a TLR4-dependent manner by bacterial PAMPs may increase macrophage cytotoxicity against tumor cells [127].

Migration of leukocytes into inflamed tissues is an early event that drives the inflammatory response. Recent studies have shown that the interaction of E-selectin with CD106 primes macrophage inflammatory response by inducing the mTORC1 axis [128]. Meanwhile, the lipid metabolite phosphatidic acid was found to efficiently stimulate the production of proinflammatory cytokines by RAW macrophages by activating the Akt/mTOR/S6K1 pathway [129]. Furthermore, other studies showed that ligation of TLR1/2 or TLR5 with bacterial peptidoglycan or flagellin, respectively, induces the PI3K/Akt/mTORC1 pathway, which upregulates the expression of NF-κB p65 and STAT3, leading to TNFα and IL-6 production by Ana-1 mouse macrophage cell line [130,131]. These findings indicate a multifaceted role of mTOR pathway in cytokine production by macrophages and its close relationship with TLR signaling.

Accordingly, TLR ligands are now widely recognized as activators of the mTOR network in macrophages [17,132], and Cot/Tpl-2/MAP3K8 kinase has been demonstrated to control mTORC1-dependent mRNA translation and cytokine production by TLR-activated macrophages [133]. Further existing data suggests that TLR activation triggers mTORC1 signaling via phosphorylation of mTOR kinase and mTORC1 substrates as well as reduction in the inhibitory effect of the TSC complex [41]. Recently, the sterol regulatory element binding protein-1 (SREBP1) has been shown to be a major downstream effector of the TLR4-mTORC1 pathway modulating pathogen phagocytosis in macrophages [134]. Importantly, mTOR signaling is also involved in TLR4-triggered synthesis of IFNγ by human and mouse macrophages [135]. In addition, Lamtor5, a component of Ragulator involved in anchoring mTORC1 to the lysosomal membrane, has been demonstrated to couple with TLR4, facilitating its trafficking to autolysosomes, which disturbs mTORC1 activation upon LPS stimulation, and thus promotes autophagic degradation of TLR4 [136]. Later, surfactant protein A was also found to promote mTORC1-driven degradation of TLR4 in LPS-treated alveolar macrophages [137]. This constitutes a clinically relevant mechanism that prevents proinflammatory overactivation of macrophages. mTOR pathway was found to block TLR2-dependent release of IL-23 in an in vitro model of human monocyte-derived macrophages infection with *Mycobacterium tuberculosis* [138]. Strikingly, IL-27 has been shown to activate PI3K/Akt/mTOR cascade to inhibit IFNγ-driven autophagy, which impairs the elimination of intracellular mycobacteria in macrophages [139]. It appears that not only bacteria defend themselves in this way; sustained mTOR activation was found to be necessary for efficient viral protein synthesis in macrophages infected with cytomegalovirus [140]. These findings suggest that pathogens may modulate mTOR signaling in macrophages to avoid the immune response, and subsequent studies confirmed this hypothesis [141].

#### 2.3.2. mTOR and Macrophage-Mediated Trained Immunity

In this regard, it is also worth noting that mTOR pathways are closely linked to trained immunity [142,143]. Along these lines, the Akt/mTOR/HIF-1α pathway induced by β-glucan promotes aerobic glycolysis in macrophages, which underlies the metabolic reprogramming characteristic of trained immunity [144]. A similar effect is induced by an active flavonoid compound, i.e., Oroxylin A [145] and by complement C5a receptor (C5aR) ligands [146]. Interestingly, mTOR-driven glycolysis feeds pyruvate into the tricarboxylic acid cycle, producing acetyl-coenzyme A, whose metabolite mevalonate in turn activates the PI3K/mTOR pathway, creating a positive feedback loop typical for long-term trained immunity [41]. Otherwise, while some studies reported that hypertonic saline solution may inhibit mTOR activity [117], high-salt conditions have recently been shown to promote macrophage-trained immunity in an mTORC1-dependent manner [147]. These observations clearly link nutritional status to the macrophage immune responses via mTOR, as reviewed in detail elsewhere [148,149], and provide new evidence that a healthy diet supports the proper functioning of the immune system. Accordingly, a low-protein diet was shown to protect against liver injury in mice infected with *Salmonella typhimurium* through reduction in mTOR signaling in macrophages, which increased their phagocytic and autophagic activities [150]. This is of crucial importance because *Salmonella* can amplify mTOR signaling in infected macrophages to block autophagic elimination of bacteria [151]. Similarly, amino acid starvation reprograms tumor-associated macrophages to downregulate mTORC1 signaling, which increases Myc expression and promotes the phagocytosis of cancer cells [152]. Also, curcumin suppressed mTORC1 activity, skewing THP-1 cells towards resting macrophages [153]. Interestingly, the contrasting results of studies on the role of mTOR pathways in regulating macrophage activation and polarization in the metabolic syndrome seem to result from differences in the composition of the diets used [59], which further emphasizes the importance of nutrients in immunomodulation of macrophage functioning. Accordingly, the metabolic effects of mTOR pathways in macrophages have been reviewed in detail by Covarrubias and colleagues, who reiterated that metabolic changes associated with mTOR pathways are context-dependent [154]. Aside from that, mTOR modulation in cardiovascular disorders may both ameliorate and exacerbate macrophage-driven inflammation [155].

#### 2.3.3. mTOR as Key Responder to Metabolic and Environmental Stimuli

Former research revealed that leptin signaling activates the mTORC1 pathway, promoting the formation of lipid bodies, where 5-lipoxygenase localizes to synthesize leukotriene B4, which additionally triggers macrophage proinflammatory activation [156], having a significant impact on the obesity-related pathophysiological conditions [157]. On the other hand, however, globular adiponectin has been shown to induce ROS and NO production by macrophages in an mTOR-dependent manner [158], even though adiponectin promotes M2 macrophage polarization [159]. Conversely, resistin was found to induce autophagy in alveolar macrophages by increasing AMPK and thus decreasing mTOR signaling [160]. Of note, molecular studies revealed that elevated levels of insulin and LPS from gut bacteria induce IL-10 in adipose tissue macrophages in an mTOR-dependent fashion to support the control of postprandial glycemia [161]. Interestingly, chronic stress induces an mTOR-driven metabolic shift in monocytes that resembles the metabolic pattern characteristic of trained immunity [162]. Similarly, oxidative stress impacts glucose consumption and lactate production by macrophages in an mTOR-dependent fashion [163], while adipose mesenchymal stromal cells seem to reprogram macrophages towards an M2-like phenotype, involving increased lipid droplet formation [164]. Moreover, mTORC1-triggered translation of proinflammatory genes has been shown to underlie the age-dependent priming of microglia [165]. In parallel, bisphenol A, an industrial chemical pollutant, impairs macrophage autophagy by promoting mTOR signal transduction pathway, which leads to their apoptosis [166], whereas rat exposure to beryllium sulphate was found to induce pulmonary inflammation due to macrophage mTOR signaling [167]. These results indicate that environmental and biological risk factors combined with lifestyle also modulate the effects of mTOR pathways induced in macrophages. Obviously, a similar relationship exists between the biological activity of macrophages and medications interfering with the mTOR pathway directly, like rapamycin or metformin, and indirectly, as proposed, for instance, for lovastatin [168].

Along these lines, rapamycin, a widely used immunosuppressant drug with anti-cancer activity, is the first medication that comes to mind in this regard. Its most important effects on macrophages are discussed throughout the text. However, it is worth emphasizing that rapamycin can induce both suppression and enhancement of the immune response. The contrast in observed effects is partly dependent on the timing and dosing regimen of rapamycin [169], and mechanistically depends on the activation state of targeted immune cells, macrophages especially, which provides the context for modulation of the mTOR pathway, as highlighted throughout the text. For instance, the abovementioned increase in proinflammatory cytokine production by rapamycin-treated macrophages is closely related to the potential of mTORC1 in regulating translation. Interestingly, everolimus, a rapamycin derivative, is also not free from this contrasting effect on the immune system [170].

Metformin is well known to inhibit mTORC1 activity and is suspected to induce mTORC2, both via AMPK [171]. Through this mechanism, metformin may impair macrophage-trained immunity [144]. However, some studies reported that long-lasting exposure to metformin increases the mTOR/HIF-1α pathway and anti-tumor activity in macrophages [172]. It can therefore be assumed that long-term therapy with this commonly used drug will not have a harmful effect on antibacterial and anti-tumor immunity, and the specific outcomes of metformin therapy on mTOR signaling in macrophages have recently been reviewed in detail by Jafarzadeh and colleagues [173]. In addition, metformin can also modulate macrophage mTOR signaling by reducing blood glucose concentration. Exactly, high glucose levels have been shown to increase mTOR phosphorylation in THP-1 macrophages, inducing downstream mTORC1 signaling that stimulates the NOD-like receptor protein 3 (NLRP3) inflammasome in an NF-κB-dependent manner, altogether leading to macrophage proinflammatory activation [174]. The resulting oxidative stress drives pathological reactions underlying metabolic syndrome and its individual components, such as diabetes or atherosclerosis [175]. Glucose has been shown to induce mTOR and NF-κB pathways, which synergistically promote IL-1β maturation in TAMs, likely exerting protumoral effects [176]. One can thus speculate that metformin and rapamycin may have a beneficial effect on macrophages in various inflammatory conditions. However, caution should be exercised when using rapamycin in diabetic individuals, as this drug may polarize macrophages towards the M1 phenotype, which may lead to the formation of refractory wounds due to overactivated autophagy [177,178]. Moreover, this drug may also exacerbate diabetic encephalopathy by inhibiting mTOR blockade of macrophage autophagy, as shown in a rat model of diabetes [179].

#### 2.3.4. The Relationship Between Autophagy, Nutritional Status, and mTOR Activity in Macrophages

The dynamic reciprocity between mTOR and autophagy makes mTORC1 a critical inhibitor of this process when nutrient availability is sufficient [180], and later reports indicate that the mTORC2 signaling cascade may also inhibit autophagy [181,182,183]. Importantly, autophagic degradation of proteins from organelles can be considered a source of amino acids for mTORC1 activation [184]. Thus, regulation of this process in macrophages is also an important element in modulating their differentiation from myeloid precursors and their biological functioning [37]. This has great therapeutic potential, firstly proven in atherosclerosis [185], especially because macrophages’ ability to accumulate cholesterol depends mostly on the mTOR axis driven by oxidized low-density lipoprotein (oxLDL) [186,187]. Accordingly, everolimus, an mTOR inhibitor, has been experimentally shown to induce autophagy in macrophages, thereby depleting them from atherosclerotic plaques [188]. Similar results were later obtained by suppressing mTOR in apolipoprotein E-deficient mice through either RNA interference [189] or modifying galectin-8 DNA methylation pattern [190]. Interestingly, it has been suggested that lactoferrin also produces analogous effects [191], whereas leucine acts in an opposite manner, i.e., prevents autophagy by triggering mTORC1 activity [192]. Finally, treatment of macrophages with mesenchymal stem cell-derived extracellular vesicles (EVs) decreased mTOR phosphorylation, which promoted autophagy-related amelioration of atherosclerosis in diabetic mice [193]. Conversely, AMPK-mTOR-TFEB signaling has recently been proposed to be responsible for homocysteine-induced impairment of macrophage autophagy [194]. The close association of mTOR-dependent signaling with the regulation of autophagy has also been demonstrated in non-canonical pathways of this process [195,196,197]. Although mTORC1 signaling has been proven to inhibit rather than promote autophagy, some opposing reports suggest that TSC1-deficiency-caused long-term mTORC1 activity promotes AMPKα-dependent autophagy in macrophages infected with *Mycobacterium tuberculosis* [198]. Other studies, however, clearly demonstrated that mTORC1 inhibition activates autophagy in *Mycobacterium tuberculosis*-infected macrophages to prevent pathogen spreading [199]. Nevertheless, it should be stressed that mTORC1 inhibition in macrophages coinfected with HIV promoted the growth of *Mycobacterium tuberculosis* [200]. On the other hand, viral infection stimulates the production of IFNγ, which has been suggested to activate the mTORC1 pathway and thus impair the phagocytic activity of macrophages against nonopsonized bacteria, thereby promoting secondary bacterial infection [201]. From another point of view, *Salmonella* infection promotes cholesterol accumulation by macrophages, which leads to mTORC1 activation and blockage of autophagy [202]. Accordingly, other pathogens have also been shown to evade the immune response by blocking macrophage autophagy in an mTOR-dependent manner [141,203,204,205,206,207,208], and pharmacological induction of autophagy may prevent pyroptosis and necroptosis of macrophages infected with *Mycobacterium tuberculosis* [209]. Notably, this pathogen developed a strategy to increase the influx of calcium ions into macrophages, which increases mTOR activity and prevents autophagy [210]. In this regard, specific mTOR modulation may improve vaccine efficiency [211], as discussed below. However, *Enterococcus faecalis*-derived lipoteichoic acid was suggested to trigger macrophage autophagy by limiting mTOR signaling [212]. Thus, inhibiting mTOR can have the opposite effect, depending, among others, on the complex that is blocked. Namely, mTORC2-deficient macrophages promote atherosclerosis due to excessive inflammasome activation driven by the transcription factor FOXO1 [213]. On the other hand, mTORC2-deficient macrophages favor colorectal cancer development in a mouse model of colitis [214]. In this regard, as a master regulator of cell proliferation, mTOR has attracted considerable attention as a possible therapeutic target in oncology [15], and tumor-associated macrophages represent one of the most promising cellular targets [215], because PI3K/Akt/mTOR signaling mostly promotes their pro-tumor M2 polarization [216].

As discussed below, mTORC1-supported inflammasome activation is one of the most important features of proinflammatory macrophages that allows for IL-1β processing. Surprisingly, however, some studies suggest that the synthesis of the inactive pro-IL-1β precursor is reduced as a result of mTORC1 overexpression caused by TSC1 depletion in macrophages [217]. Conversely, serine deprivation has been shown to significantly inhibit IL-1β production by macrophages due to the blockade of the mTOR pathway [218]. This finding raises the question of the role of amino acids other than arginine and leucine in the modulation of macrophage mTOR pathways. Accordingly, glutamine is well known to activate mTORC1 signaling, which also applies to macrophages [219]. Along these lines, limited glutamine availability reduces mTORC1 activity, and thus triggers autophagic catabolism and protects macrophages against lipotoxicity [220]. Another study revealed that mTOR inhibition in macrophages protects against lipotoxicity in an autophagy-independent manner, involving the reduction in mitochondrial substrate overload, which may have clinical applicability in inflammatory diseases accompanied by overnutrition [221].

#### 2.3.5. mTOR and Macrophage-Driven Inflammation and Immune Response Induction

Although some in vitro studies suggest that mTOR silencing may enhance macrophage phagocytic activity [222], rapamycin-induced mTOR inhibition abolished the synthesis of Rho-associated kinase 1 (ROCK-1) in macrophages, which significantly impaired the processes of chemotaxis and phagocytosis [223]. Moreover, it has been shown that newly synthesized pterocarpanquinones affecting the mTOR cascade also reduce the phagocytic activity of macrophages [224], similarly to rapamycin treatment [225] and Rheb1 deficiency [44]. These findings are in line with the results of proteomic analysis, which revealed the importance of mTOR signaling in the sustained phagocytosis of tumor cells by macrophages [226]. Interestingly, mTORC1 appears to drive macrophage phagocytic activity at higher tissue pressures accompanying inflammation [227]. Moreover, mTOR cascades modulate the formation of phagolysosomes [228] as well as are involved in macrophage engulfment of circular RNA (circRNA) [229], but appear to reduce the lysosomal activity [230]. Analogous observations were made in studies on phagocytosis of recombinant proteins of *Mycobacterium tuberculosis* [231], suggesting that stimulation of the mTOR pathway may have an adjuvant effect, enhancing the induction of an immune response. This hypothesis has recently been confirmed by demonstrating that peptidoglycan-triggered mTOR/HIF-1α pathway upregulation induces macrophage-trained immunity against *Staphylococcus aureus* after experimental vaccination [232]. At the same time, a newly developed variant of the mucosal recombinant Bacillus Calmette-Guérin (BCG) vaccine has been demonstrated to induce mTORC2 signaling in alveolar macrophages, thereby reprogramming these cells towards a metabolic phenotype characteristic of trained immunity [233]. On the contrary, inhibition of macrophage mTORC1 by rapamycin exacerbated mouse asthma related to staphylococcal infection [234]. However, particulate matter 2.5 (PM2.5), an environmental pollutant, has been proved to exert detrimental adjuvant effect, exacerbating allergic asthma in an animal model [235], and its action was shown to synergize with *Pseudomonas aeruginosa* to suppress antimicrobial activity of alveolar macrophages by increasing mTOR signaling [236], along with promoting pulmonary inflammation due to stimulation of macrophage mTOR pathway by particular matter itself [237], resulting in autophagy impairment [238]. Conversely, some studies suggested that macrophage exposure to particulate matter stimulates autophagy by driving oxidative stress, which in turn reduces mTOR expression [239]. Aside from that, another research demonstrated the protective role of mTORC1 activation in macrophages against particulate matter-driven inflammation by reducing necroptosis and NF-κB activation [240]. Therefore, the possibility of modulating mTOR in macrophages to promote an immune response should always be considered in a context-dependent manner [241].

Along these lines, spinal cord injury in rats causes a biphasic activation of mTORC1 in microglia and infiltrating macrophages, which on the one hand may promote tissue regeneration and on the other may induce an inflammatory response, depending on other microenvironmental factors [242]. Other studies conducted in the mouse excitatory injury model of epilepsy revealed that microglial mTOR plays a neuroprotective role by preventing neurons’ injury and loss as well as preserving microglial ability to clear cellular debris [243]. The latter is of special interest because one of the most important functions of tissue macrophages is the removal of apoptotic cells. Accordingly, genetic deletion of Raptor, which blocks mTORC1 activity, significantly impairs the clearance of apoptotic bodies by alveolar macrophages [35]. Altogether, these findings indicate an important role for mTOR signaling in macrophages maintaining tissue homeostasis.

Conversely, it should be emphasized that mTORC1 signaling promotes protein translation and thus may increase the ability of macrophages to present antigens [244], which is crucial to induce the adaptive humoral and cell-mediated immunity. Moreover, mTOR signaling is involved in lysosome tubulation and trafficking, as well as major histocompatibility complex (MHC) class II secretion, which enables proper antigen processing and presentation [245]. Finally, mTORC1 is essential for Langerhans cells, which are skin-resident APCs with features of macrophages and dendritic cells, to perform their antigen-presenting functions [246].

The mechanism by which innate immune cells, including macrophages, distinguish pathogenic from non-pathogenic/commensal microorganisms remains unclear. Accordingly, some studies revealed that, in contrast to a non-pathogenic mutant strain, infection of macrophages with virulent *Legionella pneumophila* led to ubiquitination of Akt, which in turn suppressed mTOR kinase activity [247], indicating that repression of mTOR pathways supports discrimination of pathogen signatures from avirulent microbes, and may even be required to initiate the inflammatory response of macrophages against pathogens. The latter is in line with the observations implicating that proinflammatory activation of macrophages in turn induces protective mechanisms to limit excessive inflammation and that these may involve mTORC1 activity [17], which additionally causes a shift in cytokine synthesis towards IL-10 instead of proinflammatory cytokines [247]. Decreased activity of the mTOR axis facilitates the clearance of *Staphylococcus aureus* by macrophages [230]. Thus, one can speculate that activation of macrophage response to pathogenic bacteria is impossible without transient repression of mTOR signaling.

On the other hand, mTORC1 signaling appears to perpetuate the proinflammatory response of macrophages to bacterial and viral infections [58,61,248]. In addition, HIV replication in intestinal macrophages has recently been found to be mTOR-dependent [249]. Altogether, mTOR-driven macrophage switches contribute to inflammatory complications, including autoimmune responses.

#### 2.3.6. The Autoimmune Context of mTOR Activity in Macrophages

A growing body of evidence supports the involvement of mTOR pathways in modulating immune reactions to self-antigens [19]. The development of an autoimmune response begins with the presentation of self-antigen to self-reactive T lymphocytes, and macrophages can act as antigen-presenting cells (APCs), especially in (auto)inflammatory conditions [250]. Moreover, it has recently been demonstrated that macrophages are capable of antigen cross-presentation, which, in the case of autoantigens, plays an important role in maintaining immune tolerance, but in certain inflammatory circumstances can trigger an autoimmune reaction [251].

In this regard, as mentioned above, mTOR-dependent pathways play an important role in regulating the process of antigen processing and presentation by macrophages [245], suggesting a possible involvement of mTOR-related cascades in the control of the self-antigen presentation. Along these lines, the costimulatory signal produced by CTLA-4-CD80/86 interaction induces in APCs the PI3K/Akt/mTOR pathway, which leads to FOXO1 exclusion from the nucleus and inhibition of autophagy. The latter reduces the turnover of peptide-MHC class II complexes, which prevent the presentation of autoantigens to self-reacting T lymphocytes [252]. However, simultaneous inhibition of PPARγ and mTORC1 promotes monocyte differentiation towards dendritic cells, which efficiently activate Th1 and CD8+ T lymphocytes [32]. Similarly, rapamycin was shown to reverse the dexamethasone-induced disability of monocytes to activate Th1 lymphocytes, assessed by IFNγ production [253], indicating that mTORC1 signaling may prevent Th1 lymphocyte-driven autoimmune reactions, and that rapamycin has the opposite immunological effect than glucocorticoids, even though both are immunosuppressive medications.

Interestingly, decreased expression of Lamtor5, the abovementioned component of Ragulator, has recently been found in peripheral blood mononuclear cells of patients with systemic lupus erythematosus (SLE). Further experimental studies have shown that mice deficient in Lamtor5 in myeloid cells develop SLE-like pathologies, and Lamtor5-deficient macrophages exhibit a hyperresponsive phenotype resulting from uncontrolled activation of the mTORC1 pathway. Conversely, efficient Lamtor5 binding to v-ATPase limits mTORC1 activity by interfering with Rag/mTORC1 interaction at the lysosomal membrane, which alleviates SLE-like pathology in mice [254]. Furthermore, Lamtor5 favors TLR4 autophagic degradation to limit macrophage activation [136]. Taking into account that, apart from LPS, TLR4 binds a variety of damage-associated molecular patterns (DAMPs) to induce mTORC1 activity in macrophages [41], it can be assumed that Lamtor5 plays an important self-protective role by downregulating mTORC1 signaling to prevent autoimmune reactions caused by macrophage overactivation.

Furthermore, IL-1β, TNFα, and IFNγ stimulate mTORC1 activity in macrophages, and robust mTORC1 stimulation severely impairs the regenerative functions of macrophages [41,255], which additionally draws attention to the risk of triggering an autoimmune reaction in highly proinflammatory conditions. Furthermore, chronic mTORC1 stimulation caused by TSC2 deficiency promotes macrophage granuloma formation in mice, which also seems to apply to sarcoidosis patients [256,257].

On the other hand, chronic mTORC2 activation disrupts the assembly of caspase-11/caspase-1/Rab39a on the actin surrounding the phagolysosome, which blocks lysosomal acidification and thus prevents degradation of IgG-containing immune complexes. This causes FcγR signaling to maintain increased mTORC2 activity, creating a feedback loop, and to activate mTORC1, which prevents the compensatory effect of autophagy and ultimately leads to SLE-like pathology in mice [258]. Lupus nephritis is a serious complication of SLE, and some mechanistic studies suggest that mTOR signaling in macrophages drives kidney injury in chronic inflammatory conditions [259], which can be prevented by rapamycin treatment [260].

One of the first reports on the possible role of the mTOR pathway in the regulation of macrophage function in autoimmunity demonstrated the dependence of IL-10 secretion by macrophages cultured with synovial T cells isolated from patients with rheumatoid arthritis on p70 S6 kinase [261]. Further studies implied that mTORC1-induced glycolysis prompts macrophages to produce IL-1β in a zinc-enriched cytoplasmic environment, aggravating the pathogenesis of rheumatoid arthritis [262]. On the other hand, mTORC1 has been proposed to mediate TNF-induced IL-10 expression and the STAT3-initiated cascade in synovial macrophages [263], whereas pharmacological blockage of Cot/Tpl-2/MAP3K8 in macrophages has been suggested to induce therapeutic effects in various autoimmune disorders [264].

The NLRP3 inflammasome, induced by PAMPs, activates inflammatory caspases to promote IL-1β processing and gasdermin D cleavage, which leads to a unique form of cell death called pyroptosis. Chronic stimulation of this signaling circuit, mostly by dysbiotic microbiota, has recently been demonstrated to contribute to the pathogenesis of various autoimmune disorders, including inflammatory bowel disease (IBD) and SLE [265]. In a mouse model of the latter disease, Li and colleagues [266] showed that mTOR networks regulate NLPR3 inflammasome activity in macrophages in an NF-κB-independent manner. Namely, simultaneous inhibition of mTORC1 and mTORC2 with INK128 prevented NLRP3 inflammasome activation by diminishing mitochondrial ROS generation. A similar inhibitory effect was observed under taraxasterol treatment [267]. In addition, mTORC1 was found to regulate pyroptosis by promoting gasdermin D oligomerization in the plasma membrane rather than cleavage upon inflammasome activation [268]. Moreover, mTORC1 appears to favor pyroptosis of synovial macrophages in inflammatory conditions [269]. These findings suggested that mTOR downregulation has therapeutic potential in autoimmune diseases by targeting the inflammasome-pyroptosis circuit. However, more selective inhibition of mTORC1 with rapamycin increased pyroptosis of macrophages infected with *Staphylococcus aureus* by reducing STAT3 phosphorylation [270], even though rapamycin was shown in other studies to reduce NLRP3 inflammasome activation in LPS-primed macrophages [267]. These discrepancies may be due to the facts that proinflammatory stimulation of mouse macrophages also activates feedback loops limiting excessive inflammation that may involve mTORC1 activity, thus explaining some of the immunostimulatory effects of mTORC1 inhibition [17], and pyroptosis is not exclusively a consequence of inflammasome activation [271], again confirming that mTOR regulation of macrophage functioning is context-dependent.

Aside from that, it is worth noting that various complications of autoinflammation, including choroidal neovascularization, appear to be potentiated by mTORC1 activity, which promotes M1 macrophage polarization [272].

Currently, therapeutic modulation of the mTOR pathway is seen as a promising strategy for inducing remission in patients suffering from autoimmune diseases, and rapamycin treatment has been first tested and has shown some beneficial effects [273]. However, experimental rapamycin treatment was found to augment the secretion of IL-12p40, IL-12p70, and IL-23 by human monocytes and macrophages stimulated with PAMPs [17]. As mentioned above, the proinflammatory effect of mTOR inhibition [132] could be explained by the observed preferential translation of IL-10 mRNA instead of IL-12 production during increased protein synthesis by mTORC1 in PAMP-stimulated macrophages [19]. Moreover, mTOR inhibition boosts MHC-complexed antigen presentation by triggering autophagy in macrophages [274], thereby increasing the risk of self-antigen presentation. These results emphasize the need for more specific and selective regulation of mTOR pathways in macrophages. In this regard, epigenetic regulation by microRNAs (miRNAs) appears to be a very promising approach.

As mentioned, the PI3Kγ/Akt/mTOR pathway blocks NF-κB activation and simultaneously stimulates C/EBPβ-dependent transcriptional program, which preserves the M2 macrophage phenotype [123], exhibiting an important clinical implication, as suggested in IBD [275]. Conversely, everolimus, the abovementioned mTOR inhibitor, was proposed to polarize macrophages towards the M2 phenotype to attenuate experimental autoimmune neuritis, the rat model of Guillain–Barre syndrome [276].

IBD is an umbrella term for Crohn’s disease and ulcerative colitis, two autoimmune disorders of the gastrointestinal tract that cause tissue damage related to inflammation, particularly in the intestines and colon. The most commonly used mouse models of IBD include 2,4,6-trinitrobenzenesulfonic acid (TNBSA)- and dextran sulfate sodium (DSS)-induced colitis, which mimic Crohn’s disease and ulcerative colitis, respectively. Dysregulated macrophage activity is a characteristic hallmark of intestinal inflammation, especially in Crohn’s disease [277,278], while modulation of mTOR pathways is gaining popularity as a promising therapy for patients with IBD [279], and IL-6 and mTORC1 signaling cascades appear to be inversely correlated [280]. Along these lines, a member of the C-type lectin receptor family, LSECtin, promotes apoptotic cell clearance by macrophages, followed by the production of growth factors for intestinal epithelium repair, which is triggered by LSECtin-induced mTORC1 activation in macrophages [281]. Furthermore, sustained mTORC1 signaling in TSC2-deficient macrophages promotes the synthesis of the polyamines (spermidine and spermine), which are uptaken by epithelial cells to reprogram their cellular metabolism towards proliferation and defense [282]. Furthermore, in certain circumstances, mTORC1 may reduce macrophage NF-κB-dependent proinflammatory phenotype and ability to activate Th1 and Th17 lymphocytes [283], which is of particular importance in the autoimmune response underlying Crohn’s disease pathology. In parallel, chronic nucleotide oligomerization domain 2 (NOD2) signaling induces characteristic macrophage hyporesponsiveness dependent on mTOR signaling, which is important in maintaining intestinal homeostasis, while NOD2-encoding gene polymorphisms increase the risk of Crohn’s disease development [284]. In a mouse model of ulcerative colitis, IFNγ signaling was demonstrated to induce mTORC1 in both epithelial cells and macrophages. Increased mTORC1 activity, in turn, inhibited the co-transcriptional action of β-catenin, which prevented epithelial DNA damage but also promoted a proinflammatory phenotype in macrophages [285], which appears to have a protective role against IBD-induced colorectal cancer [280,286]. In addition, complementary effects of mTORC1 signaling on cytokine profile of THP-1 macrophages and Caco-2 cells have been shown in in vitro co-culture studies [287]. It can therefore be assumed that mTORC1-driven macrophages ensure intestinal epithelial homeostasis and suppress autoimmune response and accompanying inflammatory reactions in IBD.

However, in other contexts, mTORC1 activity may have a detrimental effect on IBD. Accordingly, intestinal macrophage autophagy plays a significant protective role against colitis, and, as already mentioned, the mTORC1 pathway is the most prominent inhibitor of this process, while AMPK has an opposite effect [288]. Some studies have shown that the alpha7 nicotinic acetylcholine receptor (α7nAChR) plays a stimulating role in autophagy via the AMPK-mTOR circuit in macrophages [289]. Exactly, α7nAChR stimulation seems to inhibit mTORC1 in an AMPK-dependent manner, which then blocks NLRP3 inflammasome in macrophages and thus alleviates colitis [290]. In this regard, it seems reasonable to investigate the possible immunosuppressive effect of metformin in IBD, as suggested in the case of ulcerative colitis [291]. Similarly, macrophages in inflamed intestines have been demonstrated to express TREM-1 (triggering receptor expressed on myeloid cells-1), which augments their proinflammatory responses [292], likely through the mTORC1 pathway [184], whereas TREM-1 blockage restores impaired macrophage autophagic activity to suppress DSS-induced colitis in mice [293]. TREM-1 has been demonstrated to induce macrophage necroptosis by mTOR-dependent mitochondrial fission [294], implying a detrimental effect of the TREM-1–mTOR axis on macrophages in autoimmune and inflammatory contexts. On the other hand, TREM-2 has been shown to activate the Akt/mTOR signaling following microglial stimulation with beta-amyloid and apolipoprotein E (ApoE), which is expected to have a protective effect, whereas SHIP1 (SH2-domain-containing inositol 5-phosphatase 1) is a negative regulator of this pathway, resulting in aberrant microglial activation [295]. It can therefore be assumed that TREM-1- and TREM-2-induced mTOR signaling exerts opposite effects, i.e., detrimental and beneficial, on macrophage-triggered inflammation.

Furthermore, lamina propria macrophages from IL-10-deficient mice are hyperresponsive to gut microbiota, which results in colitis development [296]. Further mechanistic studies revealed that IL-10 deficiency activates mTORC1, which promotes mitochondrial ROS production and thus inflammasome activation. Conversely, physiological IL-10 signaling induces in macrophages the expression of DDIT4 (DNA damage-inducible transcript 4) in a STAT3-dependent manner, and in turn DDIT4 inhibits the mTORC1 pathway by activation of the TSC1/TSC2 complex [297,298]. The DDIT4-dependent regulatory cascade may also exert beneficial effects in diabetic wound healing by abolishing macrophage proinflammatory activation [299], and, apart from autocrine signaling, IL-10 can be delivered by other cells, including regulatory B lymphocytes, to prevent autoimmunity [300]. In the mouse model of DSS-induced colitis, EVs released by the *Taenia solium* parasite exerted an immunosuppressive effect by inducing degradation of Akt and mTORC1, thereby promoting apoptosis in macrophages [301]. Herbal compounds were suggested to inhibit macrophage-mediated inflammatory reaction in a mouse model of ulcerative colitis by downregulating PI3K/Akt/mTOR pathway [302] as well as by diminishing mTORC1-inhibitory effect on autophagy in IL-10-deficient mice that spontaneously develop colitis [303]. Interestingly, the therapeutic effect of macrophage mTOR inhibition in IBD seems to be enhanced by simultaneous repression of STAT3 signaling [304].

Macrophages play an important, yet underappreciated role in psoriatic inflammation [4], and mTOR-related signaling cascades belong to the major molecular pathways involved in its pathogenesis [305], mostly by promoting the uncontrolled proliferation of keratinocytes [306]. Therefore, one can speculate that precise modulation of mTOR signaling has an outstanding therapeutic potential by improving autophagy and interfering with cytokine-driven inflammatory reactions [307]. Interestingly, rapamycin treatment was found to reduce macrophage infiltration into the skin and draining lymph nodes of imiquimod-treated mice [308,309], whereas photobiomodulation has been shown to impact macrophage polarization via the PI3K/Akt/mTOR pathway [310], which appears to have promising implications in psoriasis phototherapy. On the other hand, sonodynamic therapy was found to promote autophagy in THP-1 macrophages by diminishing PI3K/Akt/mTOR signaling [311].

In the context of multiple sclerosis, fingolimod, an approved drug for severe relapses, has been shown to reduce Rictor expression, which in turn prevents mTORC2 activation and causes podosome amplification in mouse peritoneal macrophages, leading to increased matrix degradation and likely decreased macrophage motility [312]. Thus, the clinical significance of these findings requires further investigation. Moreover, microglia and infiltrating macrophages are involved in self-antigen presentation as well as exhibit defective autophagy, which suggests the possibility that mTORC1 drives autoimmune response in multiple sclerosis [313]. On the other hand, rapamycin treatment was shown to increase cyclooxygenase-2 secretion by LPS-stimulated rat microglia [314]. Thus, once again, mTOR pathway effects have to be considered in a context-dependent manner.

Altogether, the above-discussed immunological and biological outcomes of mTOR signaling pathways’ activation in macrophages establish mTOR kinase as a leading decision maker in macrophage fate [315] (Table 1).

## 3. Epigenetic Regulation of the mTOR Pathway in Macrophages via miRNAs—Current State-of-the-Art and Future Perspectives

The term epigenetics was proposed by Conrad Waddington in the mid-20th century [316]. Epigenetic modifications do not impact the DNA sequence but involve changes in gene expression [317], and include DNA methylation, histone modification, incorporation of histone variants, nuclear remodeling and turnover, and interference of non-coding RNAs [317,318]. Epigenetic changes in living organisms can be influenced by various factors, including both internal (developmental processes) and external (exposure to environmental factors, lifestyle) impacts [317]. Considered a major epigenetic regulator, CpG island methylation can promote or repress gene expression depending on the region [319]. Another major regulator of gene expression is histone modifications, which classically include acetylation (carried out by acetyltransferases and deacetylases), phosphorylation, and methylation. Non-coding RNAs can be divided into long non-coding RNAs (lncRNAs), small non-coding RNAs (sncRNAs), and circular RNAs (circRNAs). Among sncRNAs, we can distinguish microRNAs (miRNAs), small interfering RNAs (siRNAs), and piwi-interacting RNAs (piRNAs). These molecules affect gene expression differently; specifically, miRNAs can inhibit gene expression, while lncRNAs and circRNAs function as endogenous RNA competitors or sponges [319].

### 3.1. miRNAs

MicroRNAs (miRNAs) are a family of small (21–25 nucleotide), non-coding ribonucleic acids involved in the regulation of various biological processes by modulating gene expression [320,321]. In general, they take part in the regulation of processes, such as cell differentiation, proliferation, and apoptosis [322]. miRNAs lead to inhibition of translation, destabilization, or degradation of mRNAs by binding to the 3′ untranslated region (3′ UTR) of target mRNAs, depending on the degree of sequence complementarity [323]. Moreover, it has been described that in some cases, miRNA can also activate translation or regulate transcription [324]. Additionally, miRNA can be delivered by EVs, interfering with the function of target mRNA or miRNA antagonists (anti-miRs) [325]. According to growing scientific evidence, miRNAs emerge as crucial regulators of mTOR signal transduction in mammals [326]. It is therefore sufficient to emphasize that mTOR expression in mammals can be regulated by about 58 miRNAs that have predicted binding sites in its mRNA, 28 of which have already been confirmed in at least two of the analyzed species (Table 2).

### 3.2. Regulation of Macrophage mTOR Pathways by miRNAs

Based on the observations from the molecular studies of mouse osteoclastogenesis, showing that miRNAs play a crucial role in orchestrating the osteoclast differentiation effect in mTOR pathways [327], one can assume that macrophage differentiation in general is epigenetically regulated by miRNAs targeting mTOR-related molecules. Furthermore, since both miRNAs and mTOR signaling play crucial roles in the modulation of key cellular functions, mTOR regulation by miRNAs is critically important in the fine-tuning of macrophage activity under physiological and pathological conditions [328]. This can be achieved by miRNAs that either directly target mRNAs encoding mTOR kinase and/or other components of the mTORC1 and mTORC2 complexes or repress the translation of proteins that are up- or downstream regulators of the mTOR pathway.

Along these lines, miRNA-99a-5p is well known to directly target mTOR mRNA, and recent research proposed that this regulatory pathway may have therapeutic potential in atherosclerosis, as evidenced by overexpressing this miRNA in ApoE^−/−^ mice. Mechanistically, mTOR silencing by miRNA-99a-5p was proved to prevent NLRP3 inflammasome activation and increase autophagy in macrophages, which ameliorated atherosclerotic lesions [329]. Furthermore, increased expression of miRNA-99a in IL-4-stimulated M2 macrophages directly diminishes mTOR signaling and prompts macrophages to release miRNA-99a-containing EVs to spread M2-biasing signaling in a paracrine manner [330]. In addition to miRNA-99a, miRNA-99b is also predicted to bind mTOR mRNA, and it has been suggested to stimulate M1 macrophage polarization by enhancing NF-κB signaling due to the repressed mTOR pathway, which is of crucial importance in TAM reprogramming [331].

It should be stressed that miRNA-100 targets mRNAs encoding several components of the mTOR pathway, including mTOR itself and Raptor, and in human individuals, it has been shown to be downregulated in macrophages infiltrating fat-enriched atherosclerotic plaques [332], again confirming the role of mTOR in lipid accumulation by macrophages, as discussed above. Furthermore, mTOR downregulation by miRNA-100-5p was shown to maintain the M2 phenotype of TAMs, which was associated with increased CD206 and IL-1ra expression, resulting in tumor progression. This could be reversed by antagonizing miRNA-100 in TAMs, as shown in a mouse breast cancer model [333]. Interestingly, some studies suggest that miRNA-100-5p antagonists may also attenuate rheumatoid arthritis by restoring mTOR activity in fibroblast-like synoviocytes [334]. However, it is not clear whether the same mechanism also applies to synovial macrophages. Analogously, some data suggested that miRNA-144 may repress mTOR and thus increase autophagy in cardiomyocytes after myocardial infarction [335], but so far, there is no evidence to support a similar mechanism in macrophages and the ability of miRNA-144 to directly target mTOR mRNA. Likewise, miRNA-16-5p has been proved to induce autophagy in the tumorigenesis by downregulating the mTOR pathway [336,337], and recent observations linked this miRNA with inhibition of macrophage M1 polarization [338], which may also result from modulation of mTORC1 activity. mTOR may be directly targeted by miRNA-496 (Table 2), which has also been shown to diminish the action of Rab4A [339], the small GTPase mediating mTOR trafficking to the lysosomal membrane [340]. As an aside, mTOR docking to the lysosomal membrane is also regulated by the GTPase Rab5 [341], whose uncontrolled activation results in inhibition of mTORC1 activity [342]. Notably, Rab5 plays an important role in macrophage phagocytic activity, lipid metabolism, and foam cell formation, and its expression is also regulated by miRNAs and siRNAs [343,344]. Thus, one can assume that Rab GTPases constitute another bridge between macrophage functions and mTOR pathway modulation, which is also subject to epigenetic regulation.

Another study showed that miRNA-421-3p, enriched in EVs secreted by bone marrow-derived M2-skewed macrophages, can directly target mTOR mRNA, and thus reduce neuronal apoptosis and increase autophagy after therapeutic administration to mice with spinal cord injury [345]. Although the functional recovery was found to be mediated by mTOR modulation in neurons, an autocrine action of EV-carried miRNA-421-3p on macrophage mTOR cannot be excluded as well. In this regard, other interesting studies revealed that auto- and paracrine transfer of miRNAs via EVs between M1 and M2 macrophages could regulate ovarian aging in mice. Namely, M1 macrophage EVs were enriched in miRNA-107, whereas M2 EVs contained miRNA-99a-5p, which were shown to target PTEN and mTOR, respectively, and importantly, M2 EVs improved ovarian function in aged mice [346].

With respect to indirect mTOR modulation in macrophages, Peng and colleagues showed that EV-transmitted miRNA-467b-3p derived from endothelial cells with deleted histone demethylase UTX was taken up by macrophages, and, by downregulating PTEN, promoted the activation of mTOR signaling pathway, which led to macrophage polarization towards M2 subtype, thereby promoting neuronal recovery after spinal cord injury [347]. Similarly, miRNA-205 released by ovarian cancer cells in EVs prompted TAMs to polarize towards the M2 phenotype by diminishing PTEN expression, which augments the PI3K/Akt/mTOR pathway [348], and analogous effects have recently been attributed to miRNA-130b-3p transmitted in EVs from hepatocellular carcinoma cells [349]. Moreover, lung adenocarcinoma cell-derived miRNA-708-5p has been shown to stimulate Akt/mTOR signaling and thus PD-L1 expression by impeding PTEN expression in RAW macrophages [350]. Consequently, TAMs promote cancer progression, which implies the clinical potential of targeted therapies directed against EV-transmitted miRNAs. Other studies indicate that a similar mTOR-enhancing effect can be achieved by miRNA-21a, which inhibits the expression of PTEN and its positive regulator miRNA-200c [351]. Increased mTOR phosphorylation under the action of PTEN-affecting miR-21 has also been demonstrated in the case of arsenite-treated THP-1 macrophages [352]. LPS-triggered TLR4 signaling activates miRNA-718 production in macrophages, which, among others, was shown to target PTEN to reduce their proinflammatory overactivation in response to infection [353].

Beyond this, miRNA-200c and miRNA-144 co-deficiency promote M2 macrophage polarization after LPS stimulation, which is likely associated with altered mTOR signaling, as shown in a mouse model of nonalcoholic steatohepatitis [354].

From another point of view, miRNA-19a seems to diminish M1 polarization by activating AMPK and inhibiting HIF-1α and Akt/mTOR signaling pathways. However, the clinical implications of these findings regarding the modulation of TAM activity remain to be elucidated [355]. Mechanistic studies on sepsis-induced acute liver injury revealed that Roquin-1 participates in miRNA loading into macrophage-derived EVs and that packaged miRNAs may, in turn, diminish macrophage M1 polarization in an mTOR-dependent manner [356]. In an analogous model, miRNA-5101 has been shown to regulate the PI3K/Akt/mTOR axis in Kupffer cells [357].

Aside from that, LPS-treated mesenchymal stem cells were found to release miRNA-150-5p-enriched EVs, which were then engulfed by neighboring macrophages and enhanced their M2 polarization, because miRNA-150-5p targeted Irs1 (insulin receptor substance 1), and subsequently downregulated the PI3K/Akt/mTOR pathway [358]. Furthermore, human umbilical cord-derived mesenchymal stem cell-derived EVs may transmit miRNA-451 directly targeting TSC1, which overstimulates mTORC1 and thus inhibits autophagy in alveolar macrophages, which has beneficial effects on acute lung injury [359]. On the other hand, trophoblast-EV-derived miRNA-377-3p specifically targets NR6A1, a transcription suppressor, which results in the overactivation of the downstream mTOR/S6K1/SREBP pathway, ultimately leading to M1 polarization of macrophages, which has a deleterious effect on the fetal cardiovascular system [360]. Beyond this, breast cancer cell-derived EVs were found to transmit miRNA-148b-3p, which has been shown to induce M2 macrophage phenotype by targeting TSC2, and thus inducing mTORC1 signaling [361].

Therefore, novel therapeutic strategies can be based on RNA interference using synthetic siRNAs. Accordingly, selective mTOR inhibition with siRNA may serve as a potential therapy to restore macrophage defense against HIV in infected individuals [119]. In contrast, another study revealed that macrophage mTOR targeting by siRNA reduced NF-κB signaling and NLRP3 inflammasome activation, which attenuated high-glucose-induced inflammation [174]. Moreover, Chen and colleagues [43] emphasized the TSC2-mTOR pathway as a key factor in the differentiation of monocytes into M2-polarized TAMs. However, downregulation of TSC2 with siRNA enhanced macrophage-promoted angiogenesis in a STAT3-dependent manner and thus stimulated tumor growth. Conversely, beneficial effects were induced by mTOR inhibition with rapamycin, which polarized macrophages towards the M1 subset. Therefore, siRNA-based therapies must be designed carefully to precisely induce the expected biological effect.

Further studies on cancer have shown that glioma cells release miRNA-25-3p-containing EVs under hypoxic conditions, and that these EVs are engulfed by macrophages, which promotes their M2 polarization, thereby supporting tumor progression. Specifically, miRNA-25-3p was shown to target PHLPP2, a critical regulator of Akt dephosphorylation, which, in turn, activates the PI3K-AKT-mTOR signal transduction pathway in TAMs. Thus, interestingly, this suggests that altering miRNA-25-3p expression and inhibiting the PI3K/Akt/mTOR pathway in TAMs may represent a new approach to glioma therapy [362]. Glioma cells were also found to release EV-transmitted miRNA-3591-3p, which activates JAK2/PI3K/Akt/mTOR and STAT3 pathways, promoting the shift of macrophages towards M2 phenotype [363]. Similarly, EV-carried miRNA-99b-3p has been indicated to stimulate M2 macrophage polarization by targeting the PPP2CA, which indirectly promotes Akt/mTOR phosphorylation in the microenvironment of drug-resistant human breast cancer [364]. Otherwise, M2 macrophage polarization can be blocked by miRNA-143-3p repressing Rictor, an mTORC2 component, and, interestingly, EVs released by breast cancer cells under hypoxic conditions were shown to contain less miRNA-143-3p, which sustained M2 phenotype in TAMs [365]. Aside from that, Rictor mRNA is also targeted by miRNA-192-5p, which leads to the overactivation of hepatic M1 macrophages that drive lipotoxic injury [366] as well as by miRNA-218, which upregulation in macrophages is caused by *Legionella pneumophila* infection [367]. Similarly, a recent report suggests that miRNA-183-5p transferred by pancreatic neuroendocrine tumor-derived EVs reprograms macrophages towards the M2 phenotype by targeting PDCD4 (programmed cell death protein 4), which, in turn, modulates the PI3Kγ/Akt/mTOR pathway and increases osteopontin expression [368]. Analogous effect on TAMs has been attributed to miRNA-106b in colorectal cancer [369]. Former research showed that EVs from the same cancer source also transmit miRNA-4488, which induces macrophage M2 polarization by targeting RTN3/FABP5 and thus enhancing fatty acid oxidation via the PI3K/Akt/mTOR pathway [370]. Other studies have shown that miRNA-30c can repress REDD1, a negative regulator of mTOR in TAMs differentiating in human gastric cancer. Interestingly, hypoxia reduces miRNA-30c expression in TAMs in an HIF-1α-dependent manner, thereby decreasing mTOR-driven glycolysis, which blocks the macrophage’s switch towards the M1 subtype. These results imply the possibility of introducing a new treatment strategy based on the metabolic modification of the tumor microenvironment [371]. Such a strategy can be directed at EVs released by tumor cells under hypoxic conditions, which exhibit significant potential for metabolic modulation of macrophages. For instance, tumor-derived EVs were found to transmit let-7a, which skewed bone marrow-derived macrophages towards oxidative phosphorylation and M2-like phenotype by targeting insulin-related activators of the Akt/mTOR cascade [328,372]. Conversely, miRNA-378-3p was proposed to negatively regulate M2 macrophage polarization by affecting IL-4 signaling and IL-4R/PI3K/Akt pathway [373], which appears to diminish mTOR activity. Next, under hypoxic conditions, miRNA-193a-3p blocks the expression of PPTC7 (phosphatase PTC7 homolog), which increases Akt phosphorylation, thereby activating mTORC1 to phosphorylate 6-phosphofructo-2-kinase/fructose 2,6-bisphosphatase-3 for stimulation of phosphofructokinase activity, which is followed by an HIF-1α-dependent glycolysis enhancement [374].

In the case of immune checkpoint signaling, miRNA-708-5p has been found to increase PD-L1 expression by hampering PTEN and enhancing mTOR signaling in RAW macrophages [350], while miRNA-93 has been demonstrated to reduce PD-L1 expression and M2 polarization in bovine macrophages in an mTOR-dependent manner, which impaired the maternal-fetal tolerance in dairy cows [375]. Then, miRNA-484 has been found to inhibit CD137L (4-1BBL) expression, and thus indirectly reduce macrophage mTOR signaling and viability [328,376]. Conversely, reduced miRNA-4524a-5p activity leads to the accumulation of the transcription factor TBP, which increases the expression of T-cell immunoglobulin mucin-3 (TIM3), thereby inhibiting the mTOR signaling pathway and transforming macrophages into a profibrotic phenotype characterized by increased TGFβ secretion [377]. Some data suggest that a similar effect is induced by miRNA-29c [378].

On the other hand, Yang and colleagues showed that miRNA-155-5p, by targeting Rictor, a key component of mTORC2, activates a small GTPase RhoA, which causes modifications of the actin cytoskeleton and promotes phagocytosis in alveolar macrophages [379]. Additionally, miRNA-155 has also been shown to inhibit mTORC1 by downregulating the expression of Rheb, an mTORC1 inducer, leading to the activation of autophagic clearance of intracellular mycobacteria by macrophages [380]. Other research investigating the immunomodulatory action of mycobacterial acyl carrier protein on macrophages uncovered that miRNA-155-5p targets SHIP1, which, in turn, increases the phosphorylation of Akt and mTOR in mouse bone marrow-derived macrophages [381]. This mechanism has been demonstrated to promote *Mycobacterium tuberculosis* survival [382], but, on the other hand, may have a protective effect in the case of neuroinflammation, as suggested by the aforementioned study on microglia [295]. miRNA-155 overexpression was shown to reduce mTOR and Rheb levels in macrophages, which, surprisingly, disrupted autophagy at the autophagosome-lysosome fusion step and thus exacerbated liver injury in the alcohol feeding mouse model [383]. Finally, it is worth noting that miRNA-155 promotes M1 macrophage polarization and blocks M2 switch [384], and macrophage polarization is closely related to mTOR signaling, as discussed above and summarized elsewhere [385].

Interestingly, some studies revealed that miRNA-21 and miRNA-155 expressed in macrophages drive colonic inflammation in a mouse model of DSS-induced colitis [386] by targeting, respectively, PTEN and PDCD4 [387] as well as SHIP1 [388], which are closely related to mTOR signaling, as discussed throughout the article. Thus, therapeutic modulation of these miRNAs and the mTOR pathway may be beneficial in the treatment of ulcerative colitis. On the other hand, in macrophages infected with selected pathogenic bacteria or stimulated with BCG, miRNA-155 together with miRNA-31 have been shown to target Ppp2r5a to limit autophagy in an mTOR-dependent manner [389].

In terms of mycobacterial infection, miRNA-18a was proposed to attenuate autophagy in infected macrophages by downregulating the ATM/AMPK pathway, and indirectly promoting mTOR-driven blockage of bacterial removal [390]. Conversely, miRNA-29a has been found to enhance macrophage autophagy in atherosclerosis. Namely, by targeting binding sites in PIK3CA mRNA, miRNA-29a blocks the synthesis of the PI3K enzyme subunit, thereby preventing the activation of mTOR. In parallel, this results in the macrophage switch from M1 to M2 activation phenotype, indicating the possibility of therapeutic use of miRNA-29a in atherosclerosis [391]. Similar therapeutic potential was attributed to miRNA-761 by Wang and colleagues [392], who demonstrated that this miRNA disrupts foam cell formation, reduces oxLDL-provoked IL-18 and IL-1β secretion, and simultaneously activates macrophage autophagy, possibly by affecting the mTOR-ULK1 signaling pathway. Analogously, miRNA-210 drives autophagy in M2 macrophages and promotes their protumoral activity by targeting PI3K/Akt/mTOR signaling [393], but it direct molecular targets in macrophages remain to be elucidated. In high glucose conditions, miRNA-130a-3p was found to upregulate macrophage autophagy by targeting Yin Yang 1 (YY1), a zinc finger protein from the GLI-Kruppel family, which in turn inhibits PI3K/Akt/mTOR cascade [394]. In thyroiditis mouse and cellular models, miRNA-125a upregulation was demonstrated to impair autophagy, which was, surprisingly, accompanied by inhibition of PI3K/Akt/mTOR signaling in macrophages [395]. Beyond, some studies suggest that physiological downregulation of macrophage mTORC1 by IFNγ, which increases autophagy, microbial killing, and TLR-sensitivity, can be regulated by selected miRNAs [396].

Moreover, under nutrient-rich conditions, mTORC1 translocation to the lysosome and its subsequent activation are mediated by the interaction of Raptor-associated p62 with TNF receptor-associated factor 6 (TRAF6) [397]. The expression of the latter was reported to be blocked by miRNA-146a, which impeded mTOR signaling, and thus inhibited glycolysis in mouse adipose-tissue macrophages, thereby contributing to reducing weight gain in mice fed with a high-fat diet. Additionally, an analogous effect has been induced in miRNA-146a knockout mice using rapamycin [398,399]. Additionally, miRNA-146a-3p was shown to decrease mTOR phosphorylation by targeting VAV3 (vav guanine nucleotide exchange factor 3) [400]. In the myocardial ischemia/reperfusion injury model, macrophages transfected with miRNA-19α-3p were shown to downregulate mTOR phosphorylation, which blocks their pro-angiogenic function [401].

Otherwise, glioma-infiltrating M2 macrophages have been shown to promote tumorigenesis by lowering the levels of miRNA-92a and miRNA-15a in released EVs. The absence of these miRNAs activated the PI3K/Akt/mTOR signaling pathway due to the overexpression of RAP1B and CCND1, which were not targeted by miRNA-92a and miRNA-15a, respectively, in glioma cells. Thus, one can speculate that the use of these miRNAs may therapeutically downregulate the mTOR cascade in TAMs to reverse their protumoral activity [402].

As speculated above, macrophage polarization is a tightly regulated process, and growing evidence suggests that it is also controlled by miRNAs, as summarized elsewhere [403]. Interestingly, some of the miRNAs involved in macrophage polarization have already been shown to directly target mTOR. Among others, these include miRNA-99a, miRNA-495, miRNA-375 and let-7 (Table 2), while miRNA-146a, miRNA-148a, miRNA-21a, miRNA-125 and miRNA-155 can modulate mTOR pathways indirectly, as discussed above. These observations provide new opportunities to study mTOR-driven macrophage polarization in terms of its regulation by miRNAs.

Along these lines, TNFα promotes macrophage M1 polarization via mTORC1 [59], and this can be prevented by miRNA-99a that directly targets TNFα mRNA [404]. However, it can be assumed that miRNA-99a inhibits M1 macrophage polarization in a dual manner, also by inhibiting mTOR, which results in blocking the formation of mTORC1. Furthermore, miRNA-99a together with miRNA-150 were shown to repress mTOR signaling to induce a regulatory phenotype in CD4+ T lymphocytes [405]. Subsequently, it was demonstrated that regulatory T cell-derived EVs transfer miRNA-150 to dendritic cells, which induces their tolerogenic phenotype [406,407]. Finally, our studies revealed that miRNA-150 is released in EVs by suppressor T cells [408], and that these miRNA-150-enriched EVs induce immune tolerance in an antigen-specific manner, which is ensured by their coating with B1 cell-derived antibody light chains [409,410,411,412], as already proved in mouse models of contact and delayed-type hypersensitivity induced by haptens and protein antigens, including self-antigens [409,413,414,415,416]. Moreover, macrophages have been proved to constitute the primary targets for suppressor T cell-EV-carried miRNA-150 [413,414,417], and miRNA-150 not only induces their tolerogenic phenotype, but also prompts macrophages to multiply this signal by releasing miRNA-150-transmitting EVs expressing MHC class II [414]. These observations indicate that EV-carried miRNA-150 may induce self-tolerance. EVs protect miRNAs against degradation and provide their selective transfer to desired acceptor cells [418,419,420]. Therefore, they have recently emerged as promising candidates for the delivery of miRNA and siRNA-based therapeutics that can even be administered orally [421]. Notably, since both miRNA-150 and miRNA-99a have been proven to target mTOR and mediate immune tolerance, we have undertaken current studies to investigate the role of miRNA-based epigenetic regulation of mTOR in macrophages in an autoimmune context, including mouse models of Crohn’s disease and psoriasis.

Although epigenetic regulation via miRNAs is an important modulator of biological processes, introducing miRNA-based therapies into clinical practice is currently extremely difficult due to remaining gaps in basic knowledge and the lack of standardized handling protocols. Furthermore, as is clearly demonstrated by the regulation of mTOR expression by miRNAs, the effects of such approaches are highly context-dependent and, as yet, difficult to clearly define in clinical settings. Therefore, continued basic research in this area is incredibly valuable.

Furthermore, future studies should also focus on the metabolic regulation of miRNA biosynthesis in macrophages [398], and mTOR comes to the fore in this concept. Intriguingly, mTORC1 has been found to induce Drosha degradation through its Mdm2-dependent ubiquitination [422], suggesting that mTOR is a potent controller of miRNA synthesis. Accordingly, such an inhibitory effect of mTOR has already been observed in the case of biogenesis of several miRNAs, including miRNA-1, miRNA-21, miRNA-125b, and miRNA-143 [326]. Therefore, mTOR and miRNA signaling constitute an elegant example of a reciprocal regulatory loop that, among others, modulates macrophage functions in a context-dependent manner.

## 4. Conclusions

As members of the MPS, macrophages are involved in the clearance of circulating molecules from the blood and lymph. Therefore, they have attracted attention as the bystander targets of systemically delivered therapeutics, including miRNAs and siRNAs, especially those protected by EVs or nanoparticles. On the other hand, however, such targeting can induce multiple beneficial effects [423], making macrophages a promising target for tailored therapies for various inflammatory and (auto)immune diseases. This is of particular interest because the antigen-presenting capacity of macrophages can be exploited when manipulating miRNA-EV-based therapeutics to deliver them to target cells in an antigen-specific manner, even after administration via the oral route [325]. Moreover, specifically targeted macrophages have been proposed to multiply the therapeutic effects of EV-carried miRNAs, which seems to have great clinical applicability.

Altogether, epigenetic regulation seems to be the therapeutic strategy of the future. Therefore, further studies are needed to investigate the clinically relevant mechanisms of regulation of the mTOR pathway in macrophages via miRNA molecules. However, modulating the mTOR pathway in macrophages produces effects that are strictly dependent on the immunological, microenvironmental, metabolic, and other factors. Therefore, when attempting to modulate these pathways therapeutically, the broad immunological context and other specific conditions should always be taken into account. Moreover, this strongly implies that therapeutic approaches based on epigenetic regulation would involve a combination of cooperating miRNAs rather than a single miRNA/siRNA molecule, and these miRNAs would be protected by natural EVs designed to specifically target macrophages.

## Figures and Tables

**Table 1:** The comparison of mTORC1 and mTORC2 characteristics with a special focus on macrophage functions.

Characteristics	mTORC1	mTORC2
Composition	mTOR, Raptor, mLST8, DEPTOR and PRAS40	mTOR, Rictor, mLST8, DEPTOR, mSIN1 and Protor
Intracellular localization	mainly in lysosomal membrane	mainly in plasma membrane
Response to rapamycin	high (fast and effective)	low (after long exposure)
Main upstream regulators	growth factors, hormones, amino acids (mostly arginine and leucine, but also glutamine), cellular stress (reactive oxygen species, DNA damage), energetic substrates (glucose especially), TLR ligands, cytokines	growth factors, insulin, phosphatidylinositol-3,4,5-triphosphate, increased plasma membrane tension, TLR ligands, cytokines
Preferred substrates	4E-BP1, S6K1, ULK1, TFEB	PKCα, SGK1, Akt
Promoted biological processes	metabolism (anabolic processes) and cell growth	cell survival and proliferation
Inhibition of autophagy	confirmed	suspected
Role of active mTOR complexes in macrophage polarization	promotion of M1 phenotype in acute inflammation and infectious conditions (chronic LPS stimulation in nutrient-rich conditions), under the activity of cyclic GMP-AMP synthase (cGAS) and after exposure to high concentrations of fatty acids; promotion of M2 phenotype after acute LPS stimulation, under the activity of substance P, insulin-like peptide 3 and α-ketoglutarate; inhibition of mTORC1 (e.g., with rapamycin) promotes M2 polarization	mostly promotion of M2 phenotype
Proposed therapeutic significance of modulation of mTOR complexes in macrophages	context-dependent activation of mTORC1 promotes trained immunity; inactivation of mTORC1 in turn induces autophagy and modulates macrophage polarization to stimulate antimicrobial defense and ameliorate atherosclerotic lesions; skewing TAM polarization towards M1 phenotype by context-dependent mTORC1 modulation	skewing TAM polarization towards M1 phenotype by context-dependent mTORC2 inhibition; context-dependent activation of mTORC2 supports trained immunity

**Table 2:** The conserved miRNAs and miRNA families predicted to bind mTOR mRNA in selected mammals, based on the TargetScan database (https://www.targetscan.org/vert_80/ access date 11 June 2025).

Individual miRNA or miRNA Family	Confirmed Species
miRNA-96-5p/miRNA-1271-5p ^1^	Human, mouse, rat, cattle
miRNA-99-5p/miRNA-100-5p	Human, mouse, rat, cattle
miRNA-183-5p	Human, mouse
miRNA-212-5p	Mouse
miRNA-199-3p	Human, mouse, cattle
miRNA-150-5p	Mouse, rat
miRNA-383-5p	Mouse, rat
miRNA-101	Human, mouse, rat, cattle
let-7-5p/miRNA-98-5p	Mouse, rat, cattle
miRNA-144-3p	Human, mouse, rat, cattle
miRNA-7-5p	Human, mouse, rat
miRNA-194-5p	Mouse, rat
miRNA-217-5p	Mouse, rat, cattle
miRNA-199-5p	Mouse, rat, cattle
miRNA-122-5p	Mouse, rat
miRNA-328-3p	Mouse, cattle
miRNA-532-3p	Mouse, rat
miRNA-421-3p	Human, mouse, rat
miRNA-370-3p	Mouse, rat
miRNA-188-5p	Mouse
miRNA-324-5p	Mouse, rat
miRNA-331-3p	Mouse, rat
miRNA-362-5p	Mouse
miRNA-505-3p	Human, mouse, rat
miRNA-224-5p	Human, mouse, rat, cattle
miRNA-325-3p	Human, mouse, rat
miRNA-135-5p	Human
miRNA-375	Human
miRNA-193a-5p	Human, cattle
miRNA-129-3p	Human
miRNA-103-3p/miRNA-107	Human
miRNA-133a-3p	Human
miRNA-140-3p	Human
miRNA-128-3p	Human
miRNA-496	Human
miRNA-1306-5p	Human, cattle
miRNA-485-5p	Human, cattle
miRNA-1193	Human
miRNA-219a-2-3p	Human
miRNA-582-5p	Human
miRNA-339-5p	Human
miRNA-495-3p	Human, rat
miRNA-455-3p	Rat
miRNA-223-3p	Rat
miRNA-143-3p	Rat, cattle
miRNA-489-3p	Rat
miRNA-15-5p/miRNA-16-5p/miRNA-195-5p/miRNA-322-5p/miRNA-497-5p	Rat
miRNA-28-3p	Rat
miRNA-760-3p	Rat
miRNA-329-3p/miRNA-362-3p	Rat
miRNA-129-5p	Cattle
miRNA-302	Cattle
miRNA-134	Cattle
miRNA-758	Cattle
miRNA-296-3p	Cattle
miRNA-374	Cattle
miRNA-335	Cattle
miRNA-369-3p	Cattle

^1^ miRNA-1271-5p was shown in human and cattle species.

## Abbreviations

The following abbreviations are used without explanation in the main text of this manuscript:

DNA	Deoxyribonucleic acid
IFNγ	Interferon gamma
IL-	Interleukin-
NO	Nitric oxide
PPARγ	Peroxisome proliferator-activated receptor gamma
RNA	Ribonucleic acid
STAT	Signal transducer and activator of transcription
TGFβ	Transforming growth factor beta
TNFα	Tumor necrosis factor alpha
